# Gut microbiota patterns associated with somatostatin in patients undergoing pancreaticoduodenectomy: a prospective study

**DOI:** 10.1038/s41420-020-00329-4

**Published:** 2020-09-28

**Authors:** Guan-Qun Li, Tao Zhang, Wei-Guang Yang, Hao-Liang Zhong, Peng Xiao, Li-Wei Liu, Yong-Wei Wang, Hua Chen, Rui Kong, Gang Wang, Hong-Tao Tan, Xue-Wei Bai, Yi-Long Li, Le Li, Bei Sun

**Affiliations:** 1 Department of Pancreatic and Biliary Surgery, The First Affiliated Hospital of Harbin Medical University, Harbin, Heilongjiang China; 2 Key Laboratory of Hepatosplenic Surgery, Ministry of Education, Harbin, Heilongjiang China

**Keywords:** Pancreatic disease, Microbiology, Biomarkers

## Abstract

Postoperative pancreatic fistula (POPF) is a common and dreaded complication after pancreaticoduodenectomy (PD). The gut microbiota has been considered as an crucial mediator of postoperative complications, however, the precise roles of gut microbiota in POPF are unclear. A prospective study was developed to explore the effects of somatostatin on gut microbiota and we aim to identify the microbial alterations in the process of POPF. A total of 45 patients were randomly divided into PD group or additional somatostatin therapy group. The fecal sample of each patient was collected preoperatively and postoperatively and the gut microbiota was analyzed by 16S rRNA sequencing. Our study found that somatostatin therapy was independent risk factor for the occurrence of POPF, and it reduced the microbial diversity and richness in patients. At genus level, somatostatin therapy led to a decreased abundance in Bifidobacterium, Subdoligranulum and Dubosiella, whereas the abundance of Akkermansia, Enterococcus and Enterobacter were increased. The abundance levels of certain bacteria in the gut microbiota have significantly shifted in patients with POPF. The LEfSe analysis revealed that Ruminococcaceae could be used as microbial markers for distinguishing patients with high risk of POPF. Furthermore, Verrucomicrobia and Akkermansia could be used as preoperative biomarkers for identifying patients without POPF. Our prospective study highlights the specific communities related with somatostatin therapy and discovers POPF-associated microbial marker, which suggests that gut microbiota may become a diagnostic biomarker and potential therapeutic target for POPF.

## Introduction

Pancreatoduodenectomy (PD) is the most common procedure for malignant or benign pancreatic head tumors and periampullary tumors, which is characterized by large trauma, multiple viscera resection, long operation time and complex postoperative complications^1^. Despite of the lower postoperative mortality for the advanced surgical and post-management procedures, a significant proportion of patients develop to POPF is still high^2,3^. It has also been reported that POPF is associated with a twofold increased risk of mortality^4^. Many attempts, including either technical or pharmacologic methods, have been made to predict and prevent the incidence of POPF^5^. However, it seems to be a multifactorial event in which surgical techniques, anatomical factors, drug intervention and drainage all play crucial roles^6,7^. The perioperative inhibition of pancreatic exocrine secretion by somatostatin or its analogs has been widely used as a possible pharmacologic approach to decrease POPF. To date, many studies have explored somatostatin or its analogs as prophylaxis against POPF in patients underwent PD^8^. However, there has been no unified consensus regarding their routine prophylactic use for POPF^9,10^. Therefore, the effect of somatostatin on POPF remains to be further studied.

Human gut microbiota plays an irreplaceable role in human physiology, including metabolism, immune regulation and modulation of intestinal architecture. The interaction between the gut microbiota, immune system and intestinal barrier limits the growth of pathogenic flora, and disruption of this homeostasis leads to microbial imbalance known as “dysbiosis”^11^. Several novel studies have shown the relationship between dysbiosis of the gut microbiota and pancreatic diseases^12,13^. The clinical and fundamental scientific evidence has potentially suggested that the gut microbiota play significant roles in postoperative complications and anastomotic healing, particularly in gastrointestinal surgery^14,15^. However, the usage of microbial alterations in predicting POPF are still unclear during the postoperative clinical course of PD patients.

In our prospective study, a total of 45 patients underwent PD were enrolled and randomly divided into somatostatin therapy and non-somatostatin therapy groups. The clinical characteristics, preoperative and postoperative fecal samples were collected and the alterations of gut microbiota were analyzed. We except to identify specific microbiota which could be used for the prediction of POPF and even illustrate the substantial difference in the structure and composition of the gut microbiota. This study provides a novel insight into the feasibility of microbiota as indicators and the possibility of noninvasive diagnosis of POPF for PD patients.

## Results

### Study population

A total of 45 patients who were diagnosed with periampullary cancer were enrolled in our study. The diagnosis of periampullary tumor was based on specific imaging, such as computerized tomography (CT), magnetic resonance cholangiopancreatography (MRCP), and further confirmed by fine needle aspiration biopsy. As a result, 31 patients were enrolled in the somatostatin therapy and 14 patients were divided into control group. The incidence of POPF were monitored and clinical features were compared in different subgroups. After administration of the somatostatin therapy, 17 of the 31 patients provided additional fecal samples. In the control group, fecal samples from 8 patients were completely collected. Taken together, 50 fecal samples of 17 somatostatin therapy patients and 8 controls were included in the exploration cohort.

### Effects of somatostatin for preventing POPF

The clinical and pathological features were analyzed to identify the risk factors for POPF (Table 1). The univariable analysis result showed that pancreatic texture (*P* = 0.034) and pancreatic duct size (*P* = 0.041) was related to the POPF. Meanwhile, somatostatin therapy lowered the incidence of POPF (*P* = 0.023). The multivariable analysis indicated that somatostatin therapy was an independent risk factor for the occurrence of POPF (*P* = 0.044) (Table 2). Above all, our data revealed that prophylactic usage of somatostatin decreases the incidence of POPF.

**Table 1:** Univariate analyses of potential predictors of development of postoperative pancreatic fistula.

		ISGPF			
Variable	*n* = 45	POPF	No POPF	*χ*^2^	*P*-value
Age (years)				0.047	0.828
<60	17	6	11		
≥60	28	9	19		
Gender				0.182	0.670
Male	19	7	12		
Female	26	8	18		
BMI (median kg/m^2^)				0.044	0.833
<22	22	7	15		
≥22	23	8	15		
CA19–9				1.125	0.289
<37	20	5	15		
≥37	25	10	15		
ALb (μg/L)				1.607	0.205
<36	24	10	14		
≥36	21	5	16		
Hb (g/L)				0.711	0.399
<120	23	9	14		
≥120	22	6	16		
AST (U/L)				0.402	0.526
<59	21	8	13		
≥59	24	7	17		
ALT (U/L)				0.711	0.399
<69	22	6	16		
≥69	23	9	14		
TBIL (μmol/L)				1.607	0.205
<43	24	6	18		
≥43	21	9	12		
DBIL (μmol/L)				0.402	0.526
<21	21	6	15		
≥21	24	9	15		
ALP (U/L)				0.711	0.399
<324	22	6	16		
≥324	23	9	14		
Tumor type				0.450	0.502
PDAC	30	9	21		
Other	15	6	9		
Blood loss (mL)				0.045	0.832
<150	25	8	17		
≥150	20	7	13		
Pancreatic duct size (mm)				4.050	0.044
<3	15	8	7		
≥3	30	7	23		
Operation time (min)				2.179	0.140
<240	23	10	13		
≥240	22	5	17		
Pancreatic texture				4.500	0.034
Soft	20	10	10		
Hard	25	5	20		
Somatostatin therapy				5.184	0.023
Yes	31	7	23		
No	14	8	6

**Table 2:** Multivariate analyses of potential predictors of development of postoperative pancreatic fistula.

Variables	OR	95% CI	*P*-value
Somatostatin therapy (yes or no)	4.612	1.040–20.453	0.044
Pancreatic texture (soft vs hard)	0.533	0.121–2.347	0.405
Pancreatic duct size (<3 mm)	0.269	0.061–1.191	0.084

*Indicates that the two group have significant difference.

### Sequencing data quality assessment and OTU-based analysis

The quality of data was evaluated by the number of sample sequences in each stage of statistical data processing (Supplementary Material 1). The 16 S rRNA gene sequencing was performed for 50 fecal samples from 25 patients. A total of 5,349,772 high-quality reads were obtained and an average of 99,993 cleans tags per sample was eventually obtained. A number of 1779 operational taxonomic units (OTUs) were clustered by reads at the 97% similarity level. The final OTU list was built and the number of annotation to species tags of each level in each sample was measured. The 7 levels of taxonomy were separately presented in the raw data (Supplementary Material 2).

### Alterations in the gut microbiota of pancreatoduodenectomy

**Fig. 1:**
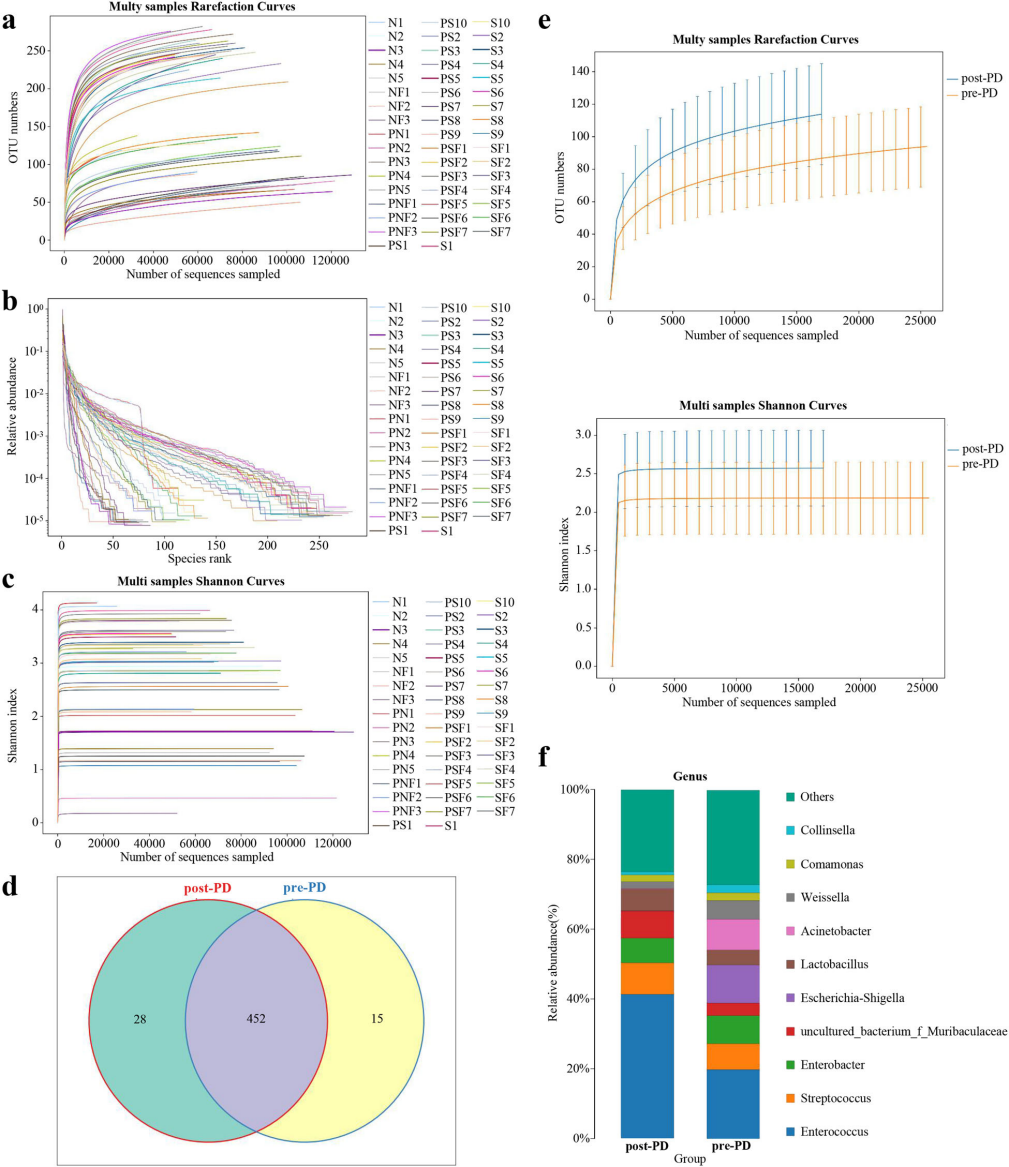
The overall species diversity of gut microbiota in different patients underwent pancreatoduodenectomy. **a** Rarefaction curve forrichness of all samples reached plateaus. **b** Rank-abundance curves showed the richness and evenness of species. The bacterial communities of themulti-samples exhibited different patterns. **c** The Shannon curve trended to be flat. **d** The Venn diagram illustrated the overlap of OTUs in gutmicrobiota. **e** Alpha diversity and richness of gut microbiota in pre-PD and post-PD patients. **f** The structural alterations and relative abundance of the gut microbiota were shown at the genus level.

The rarefaction curve was used to reflect the species diversity of samples and it indirectly reflected the abundances of the species (Fig. 1a). The curve tended to be flat with the number of sequences increased. Our data indicated that the sequencing of each sample fully reflected the species diversity. In addition, the OTU analysis results revealed different patterns of the bacterial communities of the multi samples by the rank-abundance curves (Fig. 1b). The samples reached plateaus as the number of sequences increased, which indicated that the amount of sequencing data was satisfied with reflecting the biological diversity (Fig. 1c).

To investigate the alterations in the gut microbiota in patients underwent PD, the richness and diversity of the gut microbiota was investigated. Venn diagram was constructed to evaluate the shared richness of overlapping OTU data of pre-PD and post-PD samples. Our data revealed that 452 of the 495 OTUs accounting for the total richness were common in whole samples (Fig. 1d). However, approximately 28 and 15 of the unique OTUs were identified in the pre-PD and post-PD samples. Besides, 3 common OTUs were identified preoperatively and 4 common OTUs were remained after PD surgery (Supplementary Fig. S2a). The PCoA analysis of the microbiota composition by 16 S rRNA sequencing showed that there were no major differences between pre-PD and post-PD data (Supplementary Fig. S2b). The Shannon diversity index showed that the microbial diversity of preoperative samples was lower. In addition, a trend to higher overall species richness was observed in postoperative samples (Fig. 1e). The taxon-dependent analysis was conducted to describe the composition of the fecal microbiota via the Ribosomal Database Project (RDP) classifier in different groups. The alterations of the structure and relative abundance of the gut microbiota were shown at the genus level (Fig. 1f). The PD surgery induced a decreased abundance in Escherichia-Shigella and Acinetobacter as well as an increase of Enterococcus.

### Alterations in the gut microbiota of somatostatin therapy

**Fig. 2:**
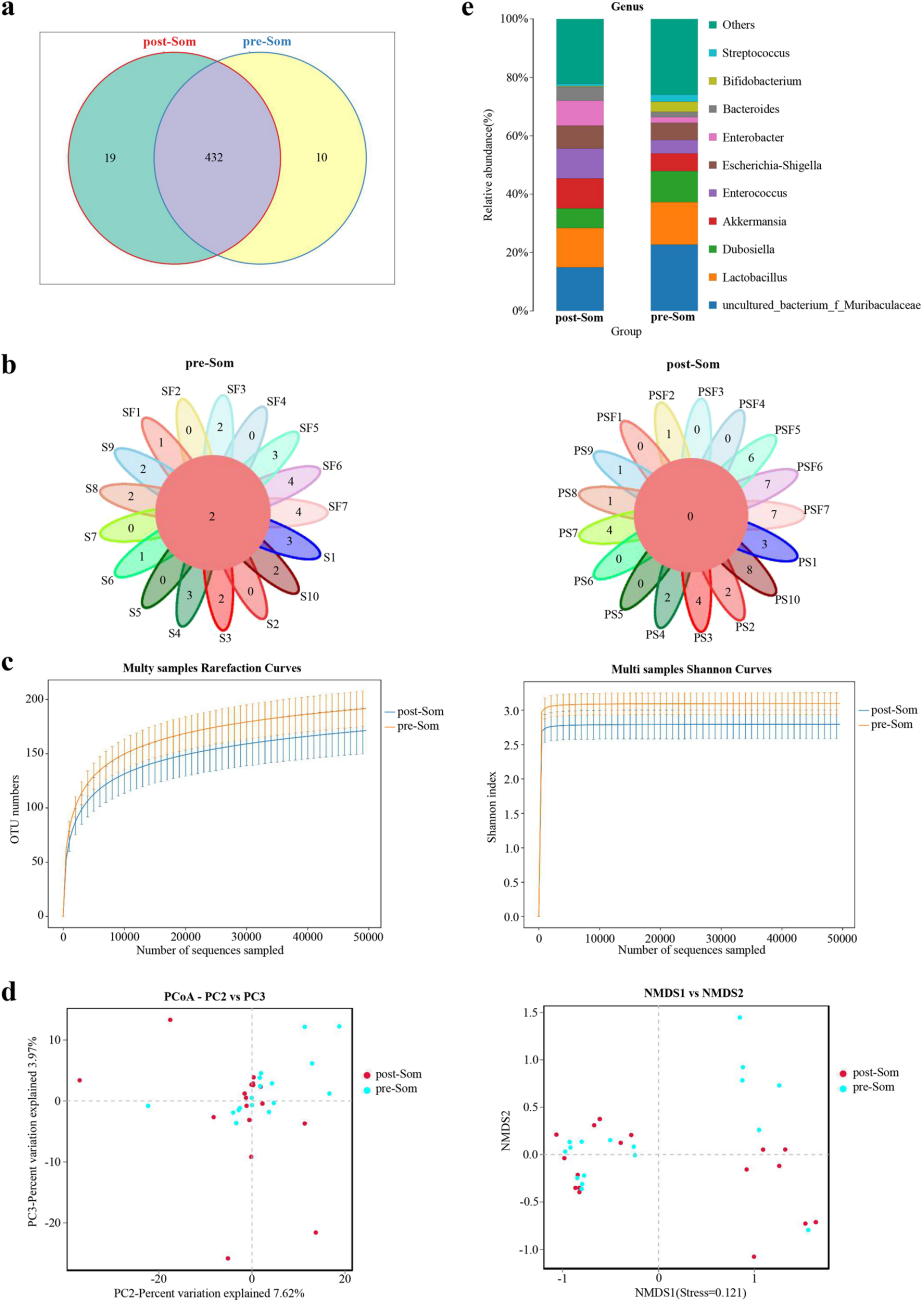
Alterations of gut microbiota in somatostatin therapy (pre-Som and post-Som) groups. **a** The common genera was showed by Venn diagram between different patients. **b** Flower Venn diagram showed the common genera. **c** Alpha diversity and richness of two group patients. **d** Each point represented a single sample, and each color represented a claster of samples. These samples were analyzed by NMDS and PCoA. **e** The abundance of gut microbiota were analyzed at genus level.

To explore the changes of gut microbiota by somatostatin therapy, a comparative analysis was conducted before and after somatostatin therapy. 432 public OTUs of intersections were publicly available from two groups (Fig. 2a). Additionally, 2 common OTUs were found in the pre-somatostatin patients and none common OTUs remained after somatostatin therapy (Fig. 2b). Remarkably, a trend to lower overall species diversity and richness in postoperative samples was observed (Fig. 2c). The microbiota composition was noted to be slightly altered between pre- and post-somatostatin patients by PCoA and NMDS (Fig. 2d). However, a simply discriminated tendency was found and it could not be transformed into significant results. The abundance changes in gut microbiota were further analyzed. At genus level, somatostatin led to a decrease in Bifidobacterium, Subdoligranulum and Dubosiella as well as an increase in Akkermansia, Enterococcus and Enterobacter (Fig. 2e).

### Gut microbial alterations in patients with or without POPF

**Fig. 3:**
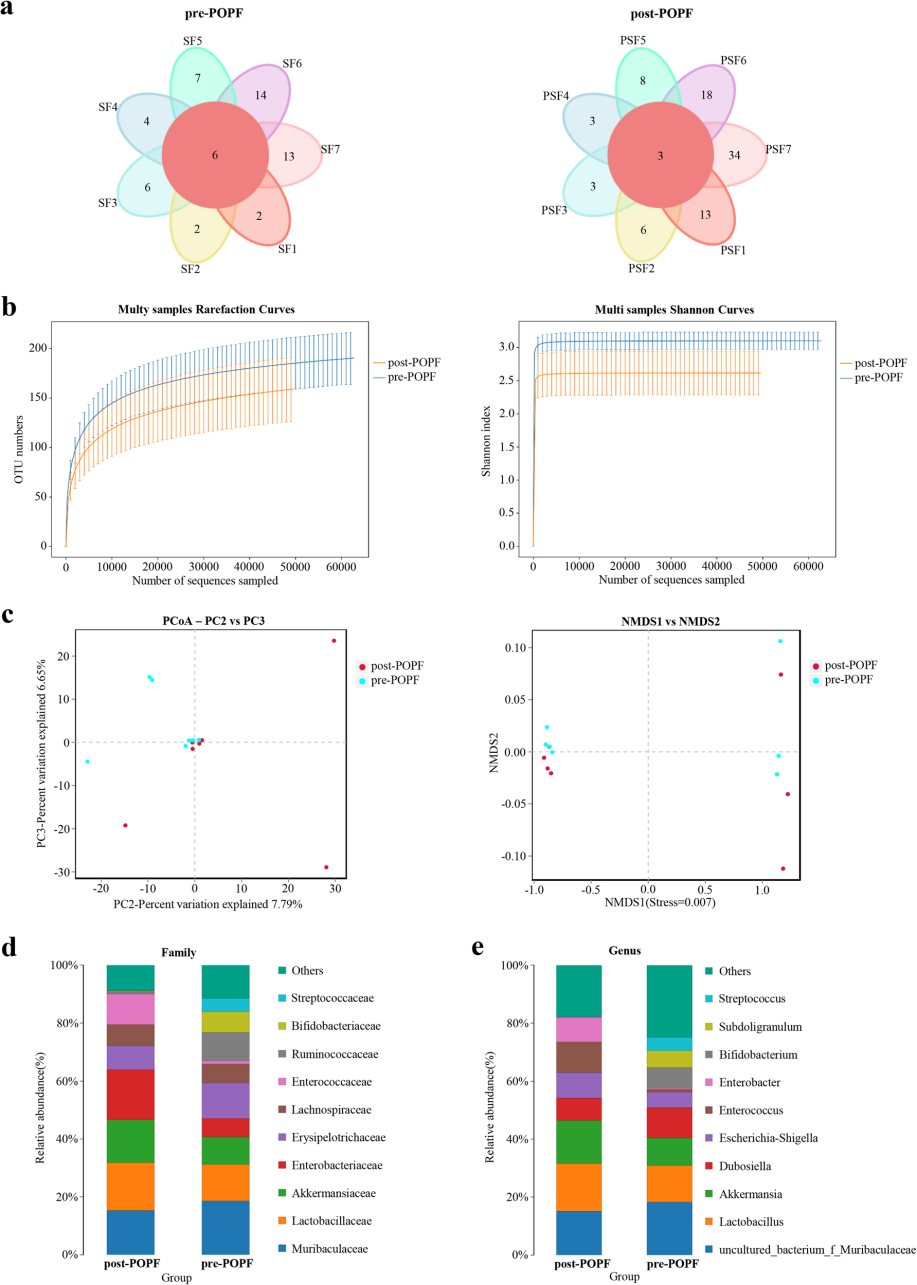
Gut microbial alterations in pre-POPF and post-POPF patients. **a** Flower Venn diagram showed the common genera between different samples. **b** Alpha diversity and richness of different patients. **c** POPF samples were analyzed by NMDS and PCoA. **d**, **e** The abundance of gut microbiota were shown at family level and genus level.

**Fig. 4:**
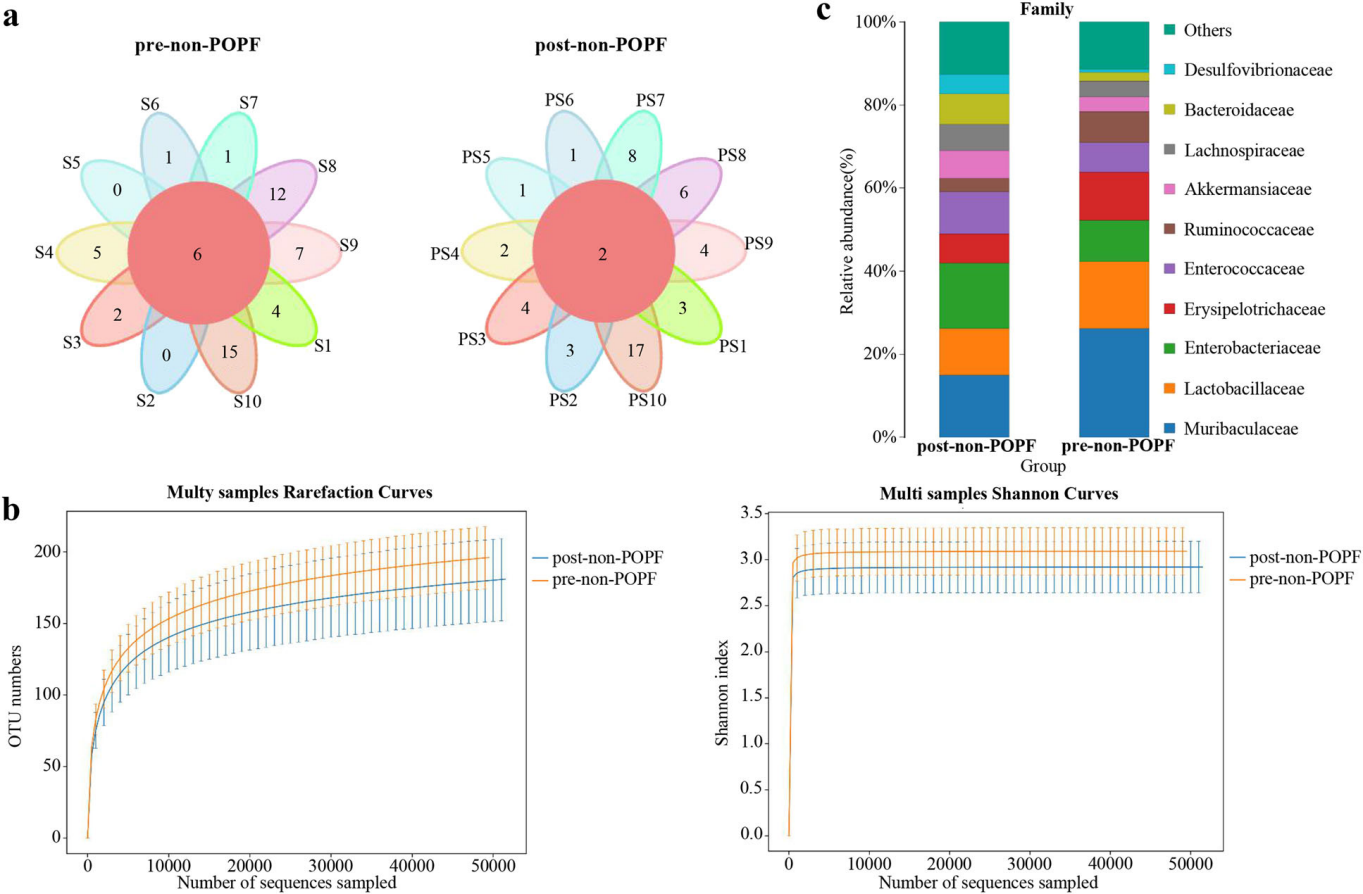
Gut microbial alterations in pre-non-POPF and post-non-POPF patients. **a** Flower Venn diagram showed common genera of patients without POPF. **b** Alpha diversity and richness of patients without POPF. **c** The alterations in relative abundance of gut microbiota were shown in family level.

**Fig. 5:**
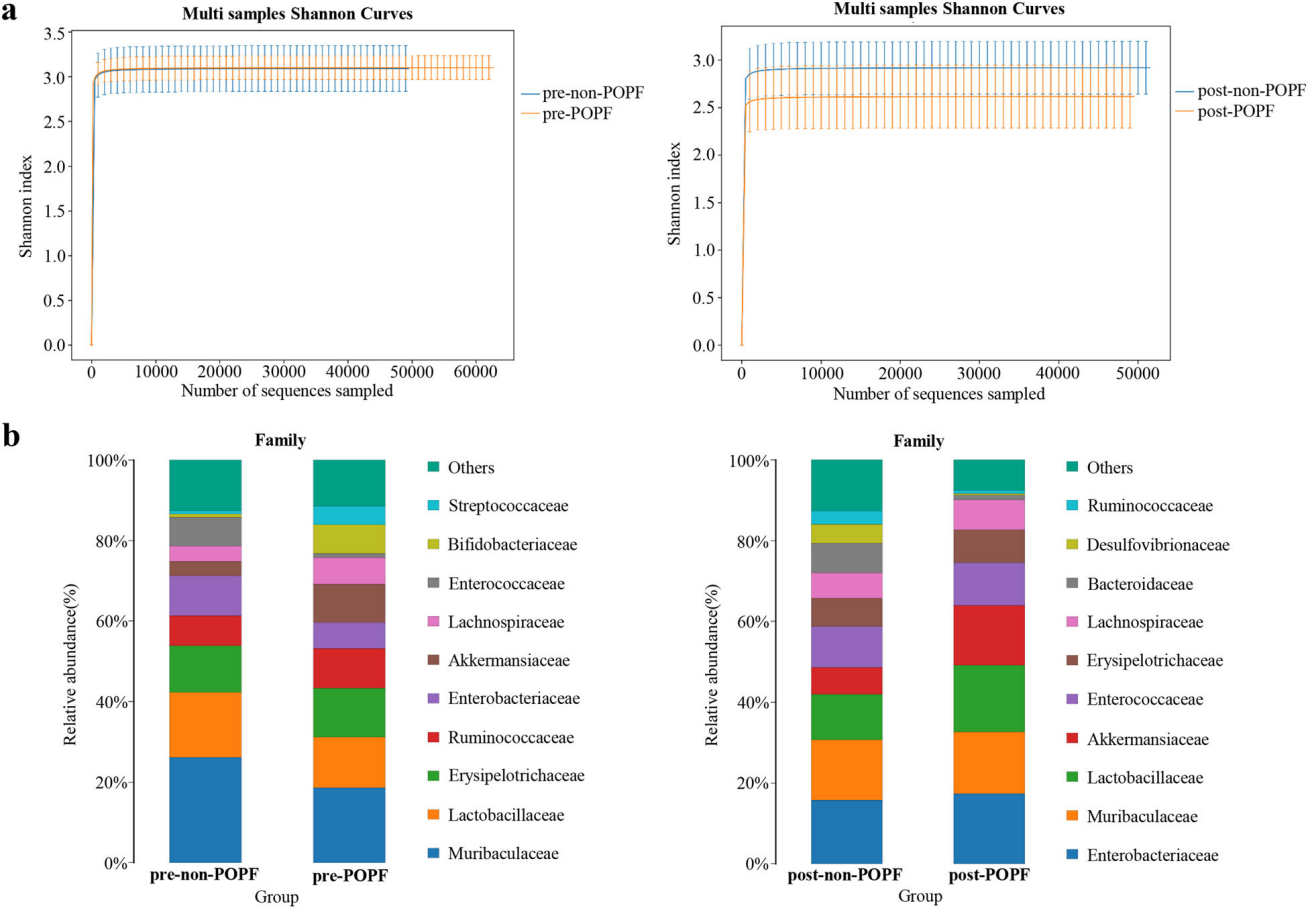
Microbial comparisons in POPF and non-POPF patients. **a** The Shannon index comparisons in patients with or without POPF. **b** The microbial abundance comparisons indicated the alterations of certain bacteria in two groups.

To investigate the specific alterations of microbiota in samples from POPF, the 7 patients with somatostatin therapy were assessed. The Venn diagram showed that 6 and 3 common OUTs were separately identified in the pre-POPF patients and post-POPF patients (Fig. 3a). The Shannon index of the post-POPF group was significantly smaller. This phenomenon was also found in the overall species richness (Fig. 3b). The weighted UniFrac method, PCoA and NMDS were performed to measure the beta-diversity values. As shown in Fig. 3c, PC2 and PC3, accounting for 7.79 and 6.65%, respectively of total variance, reflected a distribution of samples between pre-POPF and post-POPF patients. Besides, the weighted NMDS showed slight differences in bacterial composition. Notably, the proportions of Ruminococcaceae and Bifidobacteriaceae were significantly decreased, while the proportions of Enterobacteriaceae and Enterococcaceae were increased at family level (Fig. 3d). At genus level, an increase in Akkermansia and Enterococcus abundance was seen in post-POPF patients, and an increased Bifidobacterium as well as Subdoligranulum abundance was seen in pre-POPF patients (Fig. 3e). Meanwhile, the data of another 10 patients without POPF were further analyzed. Venn diagram showed that 6 and 2 common OUTs were identified preoperatively and postoperatively in patients without POPF (Fig. 4a). The trend of diversity and richness alterations in these 10 patients were similar to those patients with POPF (Fig. 4b). At the family, the proportions of Desulfovibrionaceae and Bacteroidaceae were significantly increased, while the proportion of Muribaculaceae were decreased in patients without POPF (Fig. 4c). Microbial comparisons altered between POPF and non-POPF patients were further explored. The Shannon index of the two groups was similar preoperatively. After somatostatin therapy, the Shannon index of the POPF group was smaller than non-POPF group (Fig. 5a). At family level, the proportions of Bifidobacteriaceae in POPF patients was higher preoperatively. Remarkably, the proportions of Desulfovibrionaceae and Bacteroidaceae was lower in POPF patients postoperatively (Fig. 5b). Above all, these data indicates microbial changes of certain bacteria associated with POPF.

### Predictive model between groups

**Fig. 6:**
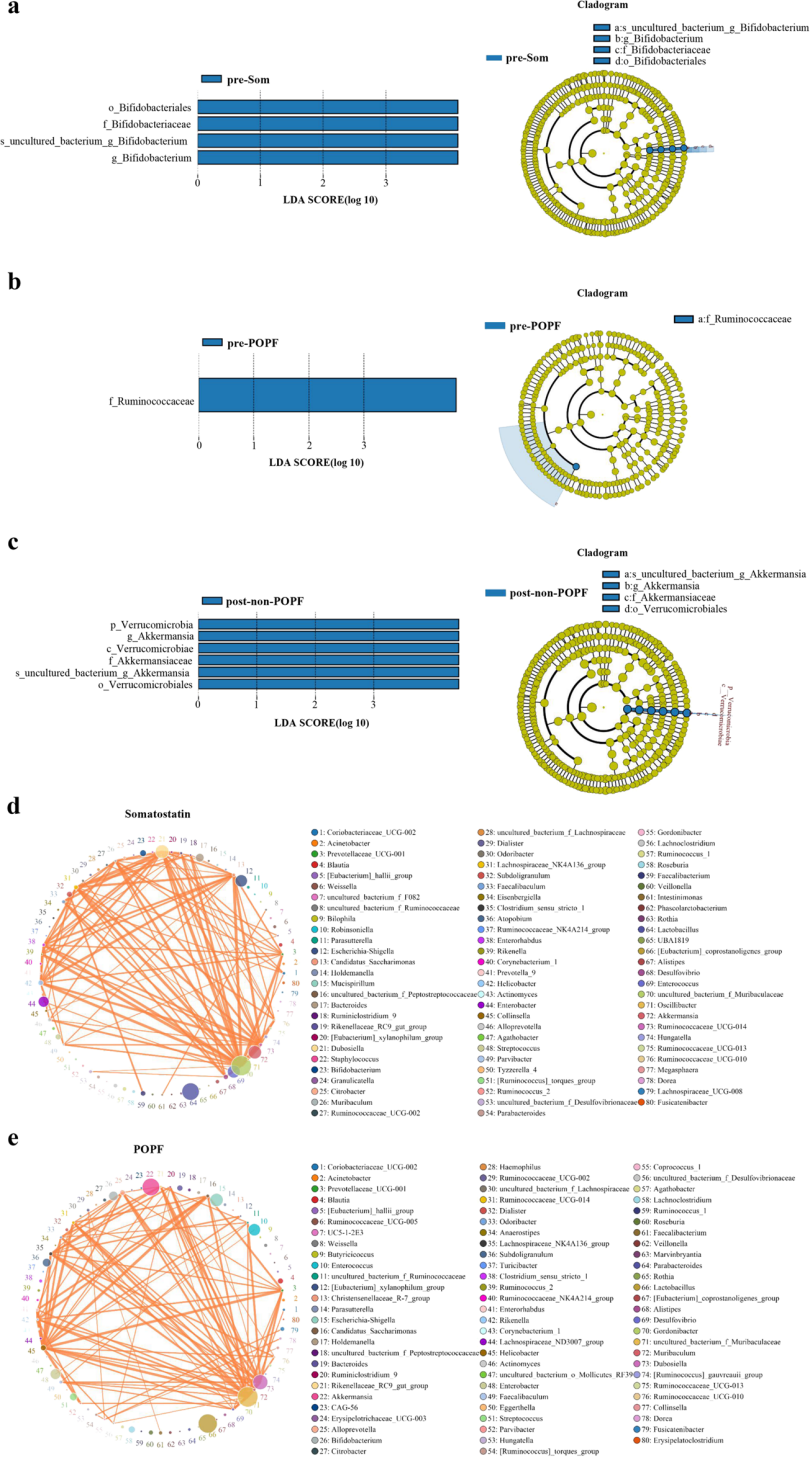
Predictive model between groups. **a**–**c** LEfSe analysis showed different abundant taxa as biomarkers by Kruskal–Wallis test (*P* < 0.05, LDA score>3.0). Cladogram representation of the different abundant taxa. The root of the cladogram represented the domain bacteria and the size of each node represents their relative abundance. No different group was labeled by yellow and significant difference were showed by blue. **d**, **e** The correlations of the relative abundance of 80 genera between somatostatin and POPF-associated gut microbial markers were showed in the interaction network. The circle size represented the abundance and the thickness of the line showed the correlated strength. The diameter of the nodes was proportional to the relative abundance. Lines between nodes denoted the correlation (*r* > 0.1 and *P* < 0.05).

To identify the specific bacterial taxa associated with different intervention, the compositions of the fecal microbiota were compared by the linear discriminant analysis effect size (LEfSe) method. A cladogram represented the structures of the fecal microbiota and the predominant bacteria. The LEfSe analysis results revealed significant differences between different groups (LDA > 3, *P* < 0.05). The LEfSe analysis was performed to determine the microbial clade differences related to PD surgery at the taxonomical level. The LEfSe analysis of microflora composition indicated no difference in species between pre-PD and post-PD patients (Supplementary Fig. S2c). In order to explore the specific communities associated with somatostatin, the compositions of the gut microbiota in pre-Som and post-Som patients were compared. Higher proportions of Bifidobacteriaceae and Bifidobacterium were found preoperatively (LDA > 3) (Fig. 6a). Furthermore, the 16S rRNA data of patients complicated with or without POPF were analyzed. Our data revealed that both of Ruminococcaceae and Akkermansia could be used as microbial markers for distinguishing POPF and non-POPF patients (LDA > 3) (Fig. 6b, c). These results confirm that microbiota can be applied for predicting POPF during the postoperative clinical practice.

We then analyzed the difference at genus level for better understanding the dynamics of gut microbiota following somatostatin treatment. These differential genera were used to construct an interaction network depicting the correlations between somatostatin and POPF-associated microbial markers. The interaction networks of somatostatin-enriched genera and POPF-enriched genera are shown in Fig. 6d, e respectively. The gut microbiota communicates to maintain dynamic equilibrium and the interactions among different genera help to understand the important roles of these species in POPF. Given evidence of the impact of microbiota on POPF, administration of specific drugs may reduce the risk of POPF through altering the gut microbiota. Our data suggest the gut microbiota as a key mediator and potential therapeutic target for POPF.

## Discussion

POPF is recognized as a life-threatening complication after PD which occurs in nearly 30% patients. It is therefore unsurprising that several procedures have been utilized to reduce its risk, such as the prophylactic administration of Somatostatin Analogs (SAs)^16^. The conception of SAs preventing POPF was proposed in 1979 and several studies have evaluated the administration of SAs for reducing the incidence of POPF after PD since then^17^. These studies have approached inconsistent views and the value of somatostatin for POPF is unclear. A prospective study was performed in our single center, and pancreatic texture, pancreatic duct size and somatostatin therapy were found to be related with POPF. Furthermore, the somatostatin therapy was discovered to be an independent risk factor for the occurrence of POPF (*P* < 0.05) in the multivariable analysis. According to the Fistula Risk Score, patient-derived risk factors of POPF included soft pancreatic texture, small pancreatic duct, high-risk pathology and excessive blood loss. Simultaneously, our study demonstrated that soft pancreatic texture and pancreatic duct size less than 3 mm are the high-risk factors of POPF. Meanwhile, our data confirmed the preventive effect of somatostatin on POPF.

The gut microbiota interacts extensively with the host by the co-metabolism of substrates and metabolic exchanges to preserve a healthy status and normal functions of the body^18^. The combination of personalized medicine and understanding about the microbiota has naturally led to identify microbial factors related to clinical outcomes. Previous studies have emphasized the significance of gut microbiota in the progression of pancreatic diseases^19^. Recent studies found that tumor resection affects the structure of the gut microbiota, which appeared to promote postoperative complications development^20^. Rogers et al. found that an increased abundance of Klebsiella and an decreased abundance of commensal anaerobes, such as Ruminococcus in postoperative fecal samples of patients with POPF^21^. Similar patterns were observed within pancreatic tissue, bile and jejunal samples. The notion that the microbiota contributes to POPF represents a novel way to be lost in thought of an old matter. Our data indicated that the variations of gut microbiota in PD patients corresponds to the progression of the POPF and somatostatin therapy. The alterations of gut microbiota imply that the identified microbial signature may be a potential strategy for predicting POPF and assessing the alterations in the gut microbiota after somatostatin therapy.

Two common OUTs were observed preoperatively and none remained after the somatostatin therapy. This alteration may be associated with the treatment of somatostatin. Besides, the overlap of OTUs in gut microbiota were found, which may aid to early intervention and identification of POPF. Furthermore, there was a trend toward higher overall microbial richness and diversity (Shannon index) in preoperative samples of PD patients. Conversely, a relative lack of diversity was observed in the gut microbiota of patients with POPF and treated with somatostatin. Previous studies have shown that the usage of antibiotics induces a dramatic reduction in the diversity of gut microbiota^22^, which is similar to our results that the gut microbial diversity is dramatically decreased after somatostatin therapy. Higher microbial diversity always links to the temporal stability of gut microbiota. Decreased gut diversity is associated with early adverse outcomes, including vulnerable resistance against invading pathogens, intestinal infections, which may result in the disruption of microbiota balance^23,24^. Thus, somatostatin and POPF change the postoperative intestinal microenvironment in PD patients, which may potentially weaken the community’s ability to resist pathogens.

In addition, the effect of somatostatin on POPF is associated with the change of microbial composition. In previous studies, the fecal samples were allocated into three different microbial communities by the structure of the gut microbiota. Schmitt et al. found that a specific community showed increased Akkermansia, Enterobacteriaceae and Bacteroidales as well as decreased in Lachnospiraceae, Prevotella and Bacteroides^25^. A microbial composition resembling the specific community were found to have a significantly higher risk for developing POPF. Comparing with samples from the American Gut Project, Rogers et al. explored disturbances of the perioperative microbiota across multiple body sites in patients underwent PD^21,26^. Postoperative samples from patients with POPF contained increased Klebsiella and decreased commensal anaerobes, including Ruminococcus. In our study, we assessed the relative abundance of taxa in patients with POPF and without POPF to investigate the specific changes of microbiota related to POPF. In accordance with previous studies, Ruminococcaceae was significantly decreased in patients with POPF, which may participate in the occurrence of POPF. Remarkably, the alterations of gut microbiota in patients without POPF may become a potential therapeutic target for preventing POPF. The significant microbial shift in patients with or without POPF will contribute to figure out the possible cause of POPF.

Many studies have demonstrated the possibility of using gut microbiota as a non-invasive biomarker^27^. To identify the specific communities related with somatostatin, the LEfSe analysis showed that abundance of commensal beneficial genera, such as Bifidobacteriaceae and Bifidobacterium, were decreased after somatostatin therapy. In recent studies^28^, the influence of Bifidobacteria on gut microbiota dysbiosis showed a potential relationship with symptoms of metabolic disorders. Zhu et al. reported the protective effects of Bifidobacteria on the pancreas were strongly correlated with those on blood glucose^29^. Somatostatin therapy induces the reduction of some dominant bacterial groups, which may aggravate the instability of postoperative state in gut microbiota. Meanwhile, Ruminococcaceae may be used as preoperative biomarkers for POPF. Similarly, other studies demonstrated that decreased abundance levels of Ruminococcus plays a crucial role in participating in POPF. Our data also indicated that both of Verrucomicrobia and Akkermansia could be used as microbial markers for distinguishing the patients without POPF preoperatively. Akkermansia muciniphila is a gram-negative anaerobic bacterium, which is the single representative member of the Verrucomicrobia phylum in the human intestinal tract. Recently, it has been considered as a promising probiotics. The pasteurized Akkermansia improves metabolic dysfunctions and the integrity of intestinal barrier and reduces plasma lipopolysaccharide levels in obese human volunteers^30,31^. Thus, the Akkermansia preparation could be supplemented properly to maintain the stable state of gut microbiota after somatostatin therapy, which may reduce the incidence of POPF. Overall, our study reveals that the changes of microbiota could serve as potential novel biomarkers for monitoring and diagnosis of POPF and evaluate the intestinal health after somatostatin therapy.

This is the first prospective study to explore the effects of somatostatin on gut microbiota composition after PD. Although our investigations attempt to provide a comprehensive insight into potential contribution of the gut microbiota related to somatostatin, several limitations are to be addressed. Firstly, our analysis showed that the incidence of POPF was 33.3%, which is slightly higher than the current published literature. Secondly, the pathological types of patients were different, which may affect the baseline of microbiota. Further studies should recruit more patients to validate the predictive power of hose biomarkers. Thirdly, the gut microbiota could be influenced by many factors during the perioperative period, such as timing of enteral nutrition, postoperative diet and antibiotics therapy. Further studies are required with multiple samples, multicenter designs and the usage of advent research techniques to discover the potential mechanisms to improve outcomes of PD patients and discover diagnostic biomarker for POPF^32^.

In conclusion, we elucidate that the prophylactic usage of somatostatin could reduce the incidence of POPF. The specific communities related with somatostatin and POPF-associated microbial markers are identified and gut microbiota may be considered as valuable biomarker for predicting POPF and evaluating the postoperative microenvironment of PD patients. Accordingly, fully understanding the mechanisms of microbial changes will bring more potentially valuable for the diagnosis and treatment of POPF.

## Materials and methods

### Patients

**Fig. 7:**
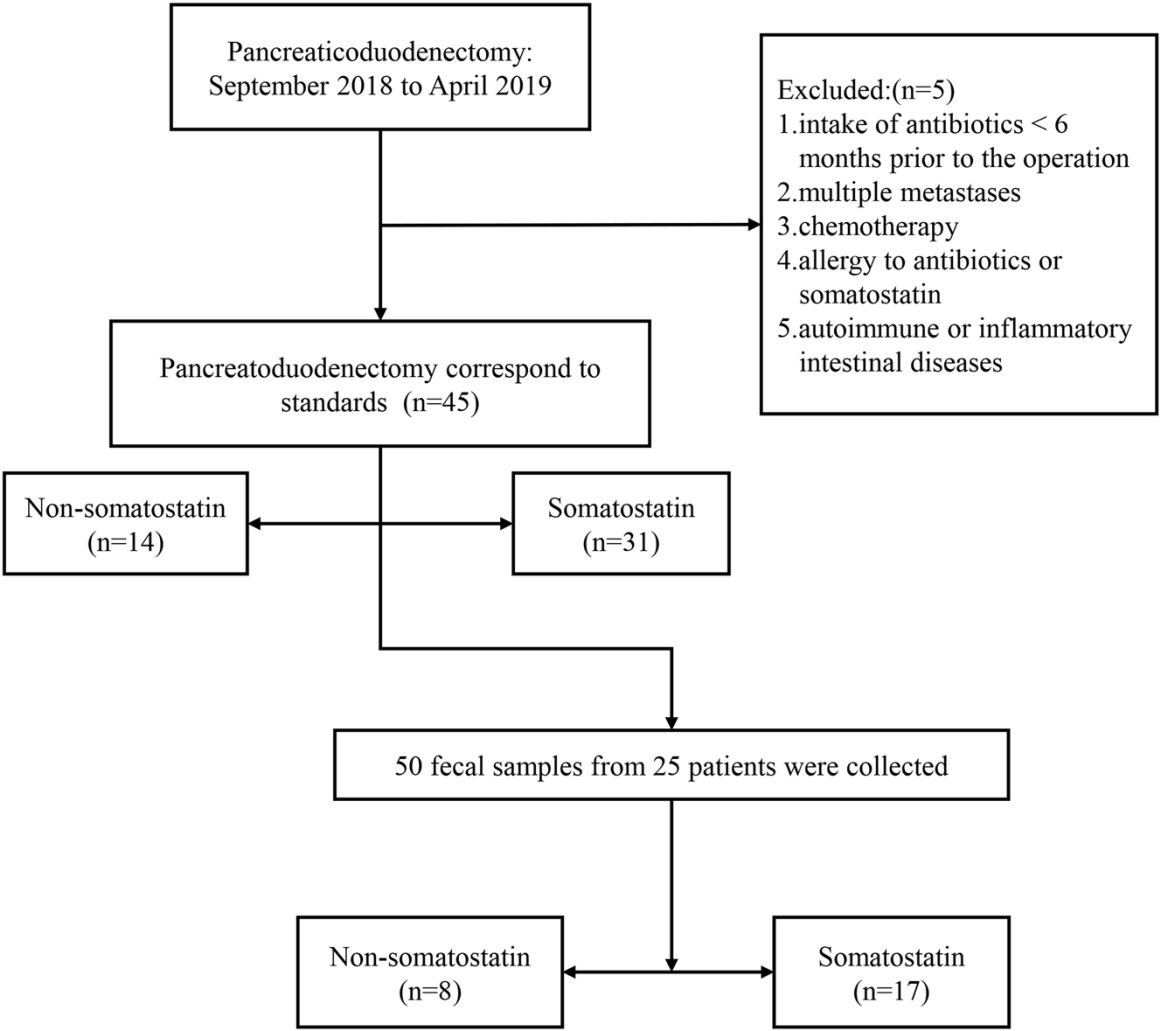
Study design and Group diagram. A total of 50 patients were enrolled and 5 patients were excluded in this prospective study. Finally, our prospective study subjects comprised 50 fecal samples from 17 patients in somatostatin therapy group and 8 patients in control group.

The prospective study subjects comprised patients underwent PD in the Department of Pancreatic and Biliary Surgery, the First Affiliated Hospital of Harbin Medical University (Harbin China) from September 2018 to April 2019. This prospective study was approved by the Ethics Committee of the First Affiliated Hospital of Harbin Medical University (China), and all participants signed the informed consent before intervention. All the data of patients were prospectively collected. A total of 50 patients were enrolled and 5 patients were excluded for intake of antibiotics <6 months prior to the operation, multiple metastases, chemotherapy, allergy to antibiotics or somatostatin, and autoimmune or inflammatory intestinal diseases. Finally, 45 (90%) were included in this prospective study, as previously described in Fig. 7. The baseline clinical characteristics of two groups had no significant difference (Table 3).

**Table 3:** Baseline characteristics in Somatostatin therapy group patients and control group patients.

Variable	Somatostatin therapy group	Control group	*P*-value
Gender, male (%)	13 (41.94%)	7 (50.00%)	0.614
Age, median years (min–max)	62.00 (38–75)	64.50 (46–73)	0.502
CA19–9, median (min–max)	47.25 (3.35–950)	42.30 (11.9–395)	0.798
Alb, μg/L median (min–max)	36.90 (28.7–43.3)	34.35 (32–44.6)	0.521
Hb, g/L median (min–max)	123.00 (84–160)	116.50 (105–135)	0.502
TBIL, μmol/L median (min–max)	158.7 (10.2–332.8)	173.5 (9.5–371.9)	0.794
BMI, median kg/m^2^ (min–max)	21.70 (17.2–29.3)	23.60 (16.6–27.9)	0.086
Pancreatic texture: hard (%)	17 (54.83%)	8 (57.14%)	0.885
Pancreatic duct size ≥3 mm (%)	21 (67.74%)	9 (64.29%)	0.820
Tumor type: PDAC (%)	21 (67.74%)	9 (64.29%)	0.820
Blood loss, mL median (min–max)	175 (100–500)	175 (100–400)	0.754

### Study design

The preoperative fecal sample was collected to determine the baseline microbiota without surgical stress, or antibiotic use, and postoperative samples were taken to evaluate the longitudinal changes in the 5–7 days after the operation. All of the fecal samples were obtained by spontaneous defecation without any manual stimulation and were collected within 3 h after defecation. The enrolled patients were randomized to either non-somatostatin intervention group (control group) or 5-day treatment with somatostatin after PD (somatostatin therapy group). The somatostatin was continuously pumped at 3 h after surgery (250 μg/h), a total of 120 h (6 mg*5 days) in post-surgery somatostatin treatment group. The clinical effects of somatostatin and occurrence of POPF will be subsequently evaluated. The diagnostic criteria of POPF follows the 2016 update of the original 2005 ISGPF definition^33^. Finally, our prospective study subjects comprised 50 fecal samples from 17 patients in somatostatin therapy group and 8 patients in control group.

### Collection of fecal samples, DNA Extraction and PCR amplification

A total of 50 fecal samples from 25 patients were collected and each sample was delivered immediately to the laboratory in an insulated box. Upon collection, the fecal samples were divided into aliquots that were then frozen in liquid nitrogen immediately and stored at −80 °C for further analysis. Microbial DNA was extracted from the feces using an PowerSoil® DNA Isolation Kit according to the manufacturer’s instructions (Supplementary Fig. S1). DNA was diluted to 1 ng/μL using sterile water. All PCR reactions were carried out with Phusion® High-Fidelity PCR Master Mix (New England Biolabs). Mix same volume of 1×loading buffer (contained SYB green) with PCR products and operate electrophoresis on 2% agarose gel for detection. Samples with bright main strip between 400–450 bp were chosen for further experiments. PCR products was mixed in equidensity ratios. Then, mixture PCR products was purified with Qiagen Gel Extraction Kit (Qiagen, Germany). The 16S rRNA high-throughput sequencing procedure was performed by commercial equipment (Illumina HiSeq PE250; Illumina, San Diego, CA, USA) in PE300 mode following the instruction provided by Biotree Co. Ltd. (Shanghai, China).

### Library preparation and sequencing

Sequencing libraries were generated using TruSeq® DNA PCR-Free Sample Preparation Kit (Illumina, USA) following manufacturer’s recommendations and index codes were added. The library quality was assessed on the Qubit@ 2.0 Fluorometer (Thermo Scientific) and Agilent Bioanalyzer 2100 system. Eventually, the library was sequenced on an Illumina HiSeq 2500 platform and 250 bp paired-end reads were generated.

### Paired-end reads assembly and quality control

Paired-end reads were assigned to samples based on their unique barcode and were truncated by cleaving the barcode and primer sequence. Paired-end reads were merged by FLASH (V1.2.7)^34^. FLASH was designed to merge paired-end reads when at least some of the reads overlap the read generated from the opposite end of the same DNA fragment, and the splicing sequences were named raw tags. Quality filtering of the raw tags was performed under specific filtering conditions to obtain high-quality clean tags according to the QIIME (V1.7.0) quality control process^35^. The tags were compared with a reference database (the Gold database) using the UCHIME algorithm to detect chimaera sequences, and the chimaera sequences were then removed^36^. The effective tags were finally obtained.

### OTU cluster and species annotation

Sequence analysis was performed using Uparse software (Uparse v7.0.1001)^37^. Operational taxonomic units (OTUs) were picked at 97% similarity cut-off. Representative sequences for each OTU were screened for further annotation. Sequencing reads were demultiplexed and filtered, and the identified taxonomy was then aligned by Greengenes database^38^. The Greengenes database was used for each representative sequence based on the RDP classifier (Version 2.2) algorithm to annotate taxonomic information^39^. To explore the phylogenetic relationships between different OTUs and dominant species in different samples (groups), multiple sequence alignment was conducted by MUSCLE software (Version 3.8.31)^40^.

### Statistical analyses

Univariable and multivariable analyses were performed to assess the risk factors for evaluating POPF by the logistic regression models. Alpha diversity was applied to analyze the complexity of species diversity in each sample based on different indices. These indices were measured by QIIME (Version 1.7.0) based on the rarefied OTU counts and were analyzed using R software (Version 2.15.3). The beta diversity analysis was used to evaluate differences in the species complexity, and beta diversity-weighted UniFrac was calculated using QIIME software (Version 1.7.0) based on the rarefied OTU counts. Principal Coordinate Analysis (PCoA) was performed to obtain the principal coordinates and to visualize complex, multidimensional data. The results of the PCoA were displayed using the WGCNA package, stats package and ggplot2 package in R software (Version 2.15.3). Differences between two groups were analyzed by R’s Vegan package. Non-metric multidimensional scaling (NMDS), an indirect gradient analysis, was performed in a 2-dimensional configuration (‘R’ vegan function ‘metaMDS’). The microbiota features differentiating the fecal microbiota were characterized by the LDA effect size (LEfSe) method, which emphasizes both statistical significance and biological relevance for identifying biomarkers. The Kruskal-Wallis rank-sum test was performed to compare the different abundance levels between assigned taxa and LDA was used to estimate the effect size of each feature. An alpha significance level of 0.05 and an effect size threshold of 3 were used as cut-off value in this study. In all statistical tests, a value of *P* < 0.05 was considered as a significant.

## Data accessibility

The raw sequences have been deposited in the Figshare and the necessary metadata can be found at https://figshare.com/. 10.6084/m9.figshare.12715202.

## Supplementary information

Supplementary material 2

Supplemental material-figure S1

Supplemental material-figure S2

Supplementary material 1

## References

[1] Peluso H, Jones WB, Parikh AA, Abougergi MS (2019). Treatment outcomes, 30-day readmission and healthcare resource utilization after pancreatoduodenectomy for pancreatic malignancies. J. Hepatobiliary Pancreat. Sci..

[2] Kimura W (2014). A pancreaticoduodenectomy risk model derived from 8575 cases from a national single-race population (Japanese) using a web-based data entry system: the 30-day and in-hospital mortality rates for pancreaticoduodenectomy. Ann. Surg..

[3] Pulvirenti A (2018). Clinical implications of the 2016 international study group on pancreatic surgery definition and grading of postoperative pancreatic fistula on 775 consecutive pancreatic resections. Ann. Surg..

[4] Vin Y (2008). Management and outcomes of post pancreatectomy fistula, leak, and abscess: results of 908 patients resected at a single institution between 2000 and 2005. J. Am. Coll. Surg..

[5] McMillan MT (2016). The characterization and prediction of ISGPF Grade C fistulas following pancreatoduodenectomy. J. Gastrointest. Surg..

[6] Zehetner J (2015). Intraoperative assessment of perfusion of the gastric graft and correlation with anastomotic leaks after esophagectomy. Ann. Surg..

[7] Rudis J, Ryska M (2014). Pancreatic leakage and acute postoperative pancreatitis after proximal pancreatoduodenectomy. Rozhl. Chir..

[8] Ma LW (2017). The cost of postoperative pancreatic fistula versus the cost of pasireotide: results from a prospective randomized trial. Ann. Surg..

[9] Adiamah A (2019). The use of prophylactic somatostatin therapy following pancreaticoduodenectomy: a meta-analysis of randomised controlled trials. World J. Surg..

[10] Allen PJ (2014). Pasireotide for postoperative pancreatic fistula. N. Engl. J. Med.

[11] Tang WH (2013). Intestinal microbial metabolism of phosphatidylcholine and cardiovascular risk. N. Engl. J. Med.

[12] Memba R (2017). The potential role of gut microbiota in pancreatic disease: a systematic review. Pancreatology.

[13] Signoretti M, Roggiolani R, Stornello C, Delle FG, Capurso G (2017). Gut microbiota and pancreatic diseases. Minerva Gastroenterol. Dietol..

[14] Bachmann R, Leonard D, Delzenne N, Kartheuser A, Cani PD (2017). Novel insight into the role of microbiota in colorectal surgery. Gut.

[15] Murphy R (2017). Differential changes in gut microbiota after gastric bypass and sleeve gastrectomy bariatric surgery vary according to diabetes remission. Obes. Surg..

[16] Zizzo M, Ugoletti L, Manenti A, Annessi V, Pedrazzoli C (2019). Pasireotide for the prevention of postoperative pancreatic fistula: an open debate. HPB.

[17] Klempa I, Schwedes U, Usadel KH (1979). Prevention of postoperative pancreatic complications following duodenopancreatectomy using somatostatin. Chirurg.

[18] Nicholson JK, Holmes E, Wilson ID (2005). Gut microorganisms, mammalian metabolism and personalized health care. Nat. Rev. Microbiol.

[19] Akshintala VS, Talukdar R, Singh VK, Goggins M (2019). The gut microbiota in pancreatic disease. Clin. Gastroenterol. Hepatol..

[20] Cong J (2018). A pilot study: changes of gut microbiota in post-surgery colorectal cancer patients. Front. Microbiol.

[21] Rogers MB (2017). Disturbances of the perioperative microbiota across multiple body sites in patients undergoing pancreaticoduodenectomy. Pancreas.

[22] Lange K, Buerger M, Stallmach A, Bruns T (2016). Effects of antibiotics on gut microbiota. Dig. Dis..

[23] Schubert AM, Sinani H, Schloss PD (2015). Antibiotic-induced alterations of the murine gut microbiota and subsequent effects on colonization resistance against clostridium difficile. mBio.

[24] Owens RC, Donskey CJ, Gaynes RP, Loo VG, Muto CA (2008). Antimicrobial-associated risk factors for Clostridium difficile infection. Clin. Infec. t Dis..

[25] Schmitt FCF (2019). Gut microbiota patterns correlate with higher postoperative complication rates after pancreatic surgery. BMC Microbiol.

[26] Feng Y (2019). An examination of data from the American Gut Project reveals that the dominance of the genus Bifidobacterium is associated with the diversity and robustness of the gut microbiota. Microbiologyopen.

[27] Zhao F (2018). Alterations of the gut microbiota in Hashimoto’s thyroiditis patients. Thyroid.

[28] Vazquez-Gutierrez P, de Wouters T, Werder J, Chassard C, Lacroix C (2016). In vitro high iron-sequestrating bifidobacteria inhibit enteropathogen growth and adhesion to intestinal epithelial cells. Front. Microbiol..

[29] Zhu G (2018). Bifidobacteria attenuate the development of metabolic disorders, with inter- and intra-species differences. Food Funct..

[30] Dao MC (2016). Akkermansia muciniphila and improved metabolic health during a dietary intervention in obesity: relationship with gut microbiota richness and ecology. Gut.

[31] Depommier C (2019). Supplementation with Akkermansia muciniphila in overweight and obese human volunteers: a proof-of-concept exploratory study. Nat. Med..

[32] Li CX, Andrew Z (2020). Application of image fusion in diagnosis and treatment of liver cancer. Appl. Sci..

[33] Bassi C (2017). The 2016 update of the International Study Group (ISGPS) definition and grading of postoperative pancreatic fistula: 11 years after. Surgery.

[34] Magoč T, Salzberg SL (2011). FLASH: fast length adjustment of short reads to improve genome assemblies. Bioinformatics.

[35] Caporaso JG (2010). QIIME allows analysis of high-throughput community sequencing data. Nat. Methods.

[36] Edgar RC, Haas BJ, Clemente JC, Quince C, Knight R (2011). UCHIME improves sensitivity and speed of chimera detection. Bioinformatics.

[37] Edgar RC (2013). UPARSE: highly accurate OTU sequences from microbial amplicon reads. Nat. Methods.

[38] DeSantis TZ (2006). Greengenes, a chimera-checked 16S rRNA gene database and workbench compatible with ARB. Appl. Environ. Microbiol.

[39] Wang Q, Garrity GM, Tiedje JM, Cole JR (2007). Naive Bayesian classifier for rapid assignment of rRNA sequences into the new bacterial taxonomy. Appl. Environ. Microbiol..

[40] Edgar RC (2004). MUSCLE: a multiple sequence alignment method with reduced time and space complexity. BMC Bioinformatics.

