# Correction: Gut microbiota patterns associated with somatostatin in patients undergoing pancreaticoduodenectomy: a prospective study

**DOI:** 10.1038/s41420-020-00339-2

**Published:** 2020-10-15

**Authors:** Guan-Qun Li, Tao Zhang, Wei-Guang Yang, Hao-Liang Zhong, Peng Xiao, Li-Wei Liu, Yong-Wei Wang, Hua Chen, Rui Kong, Gang Wang, Hong-Tao Tan, Xue-Wei Bai, Yi-Long Li, Le Li, Bei Sun

**Affiliations:** 1 Department of Pancreatic and Biliary Surgery, The First Affiliated Hospital of Harbin Medical University, Harbin, Heilongjiang China; 2 Key Laboratory of Hepatosplenic Surgery, Ministry of Education, Harbin, Heilongjiang China

**Keywords:** Pancreatic disease, Microbiology, Biomarkers

Correction to: *Cell Death Discovery*

10.1038/s41420-020-00329-4 published online 28 September 2020

In the original version of this article, Figs. 1–6 and their legends were mismatched. This has now been corrected in the PDF and HTML versions of the article.

