# Supplementary figures and images for: Gut microbiota patterns associated with somatostatin in patients undergoing pancreaticoduodenectomy: a prospective study
Source: Cell Death Discov. 2020 Sep 28;6:94. doi: 10.1038/s41420-020-00329-4 (PMC7522245; doi:10.1038/s41420-020-00329-4)

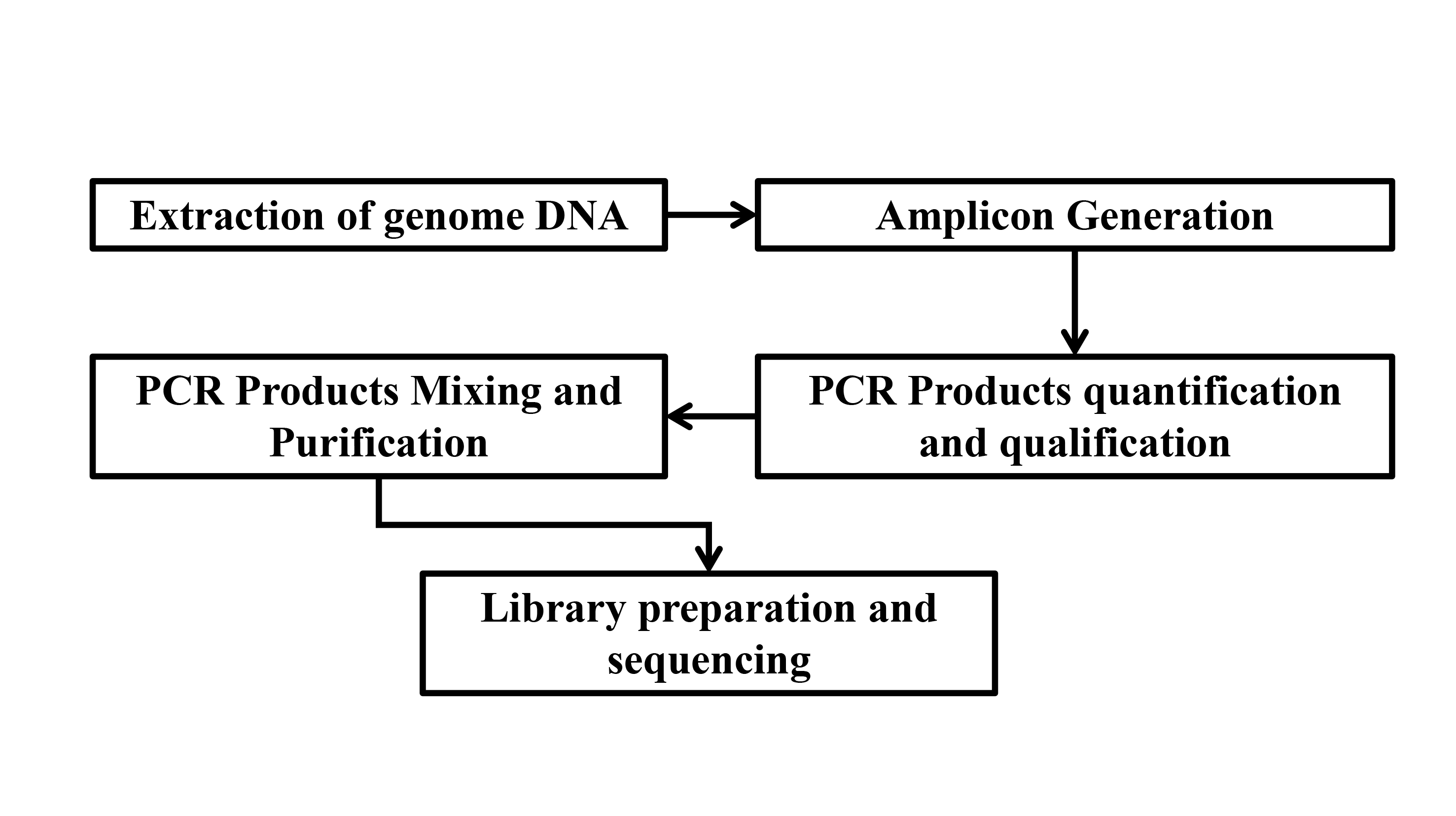

Supplement: Supplementary file 2 — Supplemental material-figure S1 [file 41420_2020_329_MOESM2_ESM.tif]

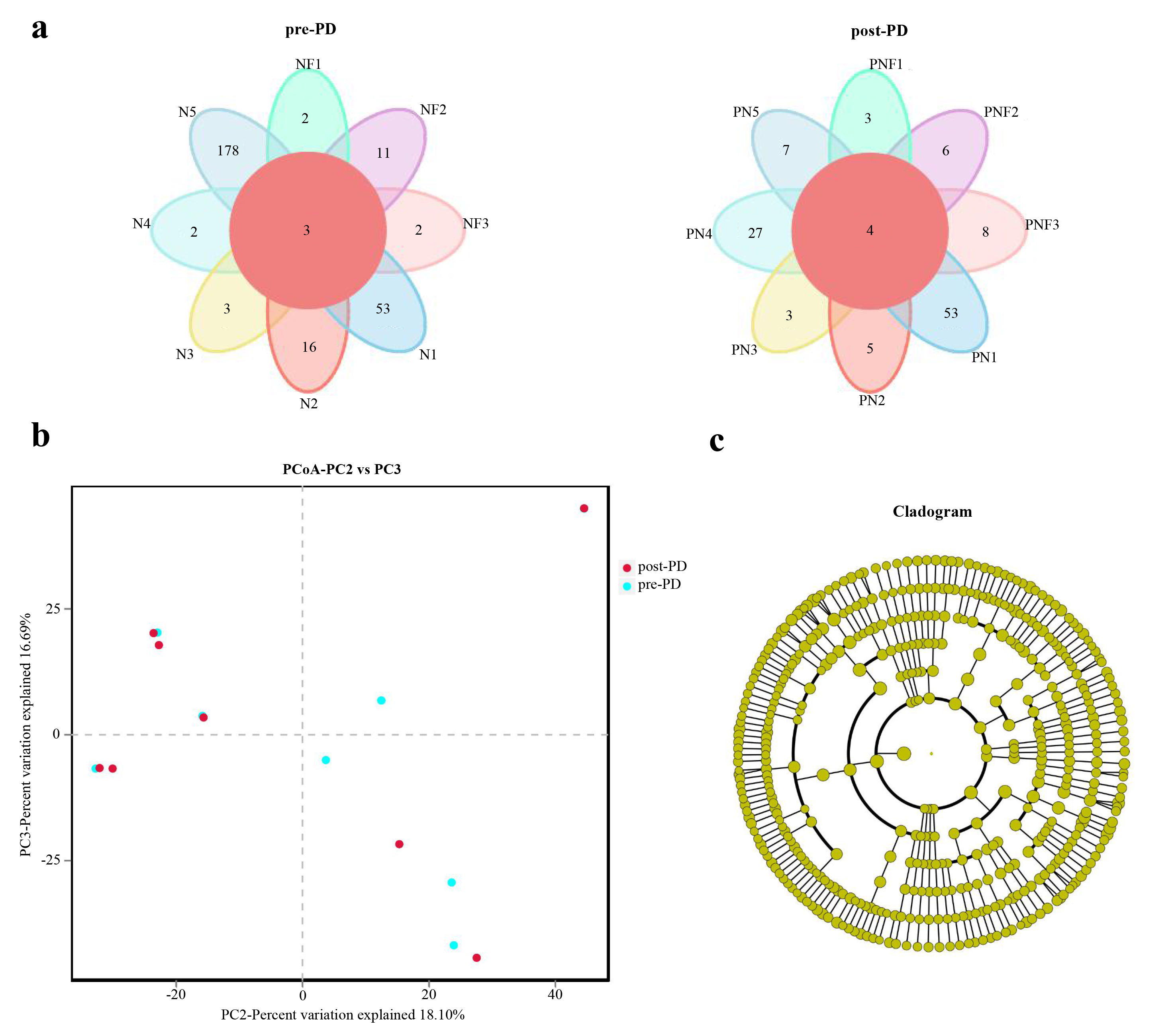

Supplement: Supplementary file 3 — Supplemental material-figure S2 [file 41420_2020_329_MOESM3_ESM.tif]
